# Spinal Cord Abnormalities in Early Pediatric Multiple Sclerosis

**DOI:** 10.1002/acn3.70046

**Published:** 2025-04-17

**Authors:** Monica Margoni, Paola Valsasina, Paolo Preziosa, Martina Rubin, Mor Gueye, Lucia Moiola, Maria Assunta Rocca, Massimo Filippi

**Affiliations:** ^1^ Neuroimaging Research Unit, Division of Neuroscience IRCCS San Raffaele Scientific Institute Milan Italy; ^2^ Neurology Unit IRCCS San Raffaele Scientific Institute Milan Italy; ^3^ Neurorehabilitation Unit IRCCS San Raffaele Scientific Institute Milan Italy; ^4^ Vita‐Salute San Raffaele University Milan Italy; ^5^ Neurophysiology Service IRCCS San Raffaele Scientific Institute Milan Italy

**Keywords:** atrophy, lesion, MRI, pediatric multiple sclerosis, spinal cord

## Abstract

Spinal cord lesions and atrophy in the cervical region are common in adult multiple sclerosis (MS) and correlate with disability. Whether similar abnormalities occur in pediatric MS patients is largely unknown. Clinical and MRI evaluations were performed in 38 pediatric MS patients and 13 healthy controls (HC). No significant differences in upper cervical cord area were found between MS patients and HC or between patients with and without lesions. Patients with lesions showed increased cord volume, co‐localizing with lesions, likely reflecting inflammation. Our results suggest that upper cord atrophy is not a prominent feature in early pediatric MS, underscoring the inflammation‐driven characteristic of these patients.

## 1 Introduction

Spinal cord lesions and atrophy, particularly in the cervical region, are common in adult multiple sclerosis (MS) patients and correlate with clinical disability [1, 2, 3]. In pediatric MS, spinal cord damage remains largely unexplored, with a few studies reporting neither significant atrophy nor microstructural abnormalities compared to healthy controls (HC) [4, 5]. Recently, three‐dimensional (3D) T1‐weighted magnetic resonance imaging (MRI) sequences have shown comparable ability to short‐tau inversion recovery (STIR) in detecting focal spinal cord lesions at 3T, with the advantage of enabling assessment of lesions, area, and volumetry with a single acquisition [6]. Using this sequence, voxel‐wise analysis of the spinal cord may allow regional detection of volumetric abnormalities, improving the understanding of MS pathophysiology [6, 7]. To date, no voxel‐wise analysis has explored the spatial relationship between focal lesions and volumetry in the spinal cord of pediatric MS patients.

This study investigated whether whole‐cord area and regional cord volumetric abnormalities can be detected in pediatric MS patients and their association with lesions and clinical disability.

## 2 Methods

### 2.1 Ethics Statement

This study involves human participants and was approved by the Ethics Committee of IRCCS San Raffaele Scientific Institute. Participants gave informed consent to participate in the study before taking part.

### 2.2 Participants

This cross‐sectional, retrospective, observational study included 38 consecutive relapsing–remitting (RR) pediatric MS patients [8] within 3 years from disease onset. Patients had to be relapse‐ and steroid‐free for at least 1 month prior to clinical and MRI assessment. The exclusion criterion was a history of other primary neurological disorders in addition to MS. Thirteen pediatric HC with no previous history of neurological dysfunction and a normal neurological examination served as controls.

### 2.3 Clinical Assessment

On the day of MRI acquisition, pediatric MS patients underwent a complete neurologic evaluation, with the rating of the Expanded Disability Status Scale (EDSS) score and the recording of ongoing disease‐modifying treatments.

### 2.4 MRI Acquisition

Using a 3.0T Ingenia MR scanner (Philips Medical System), the following sequences were acquired from all subjects during a single session: (1) Brain: (a) 3D fluid attenuated inversion recovery (FLAIR); (b) 3D T1‐weighted turbo field echo; (2) Cervical cord: (a) sagittal two‐dimensional STIR (details in the Data S1).

### 2.5 Brain MRI Analysis

Focal T2‐hyperintense white matter (WM) lesions were identified by a fully automated and validated approach using 3D FLAIR and 3D T1‐weighted images [9]. T2‐hyperintense WM lesion volume (LV) was obtained for each patient from their lesion masks, after a visual check of automatic segmentations. After refilling T1‐hypointense lesions, head size was estimated on 3D T1‐weighted scans by calculating the normalization scaling factor produced by FSL SIENAx software.

### 2.6 Assessment of Upper Cervical Cord Lesions

Lesions between the upper border of C1 and the inferior border of C3 level were analyzed. T1‐hypointense lesions were consensually identified on the 3D T1‐weighted scans by two physicians blinded to clinical data, with sagittal STIR images aiding in lesion confirmation and counts. Upper cervical cord T1‐hypointense lesions were contoured using a semiautomated method implemented in Jim 7.0 (Xinapse Systems Ltd., Colchester, UK), and corresponding lesion masks were created.

### 2.7 Upper Cervical Cord Area Assessment

**FIGURE 1:**
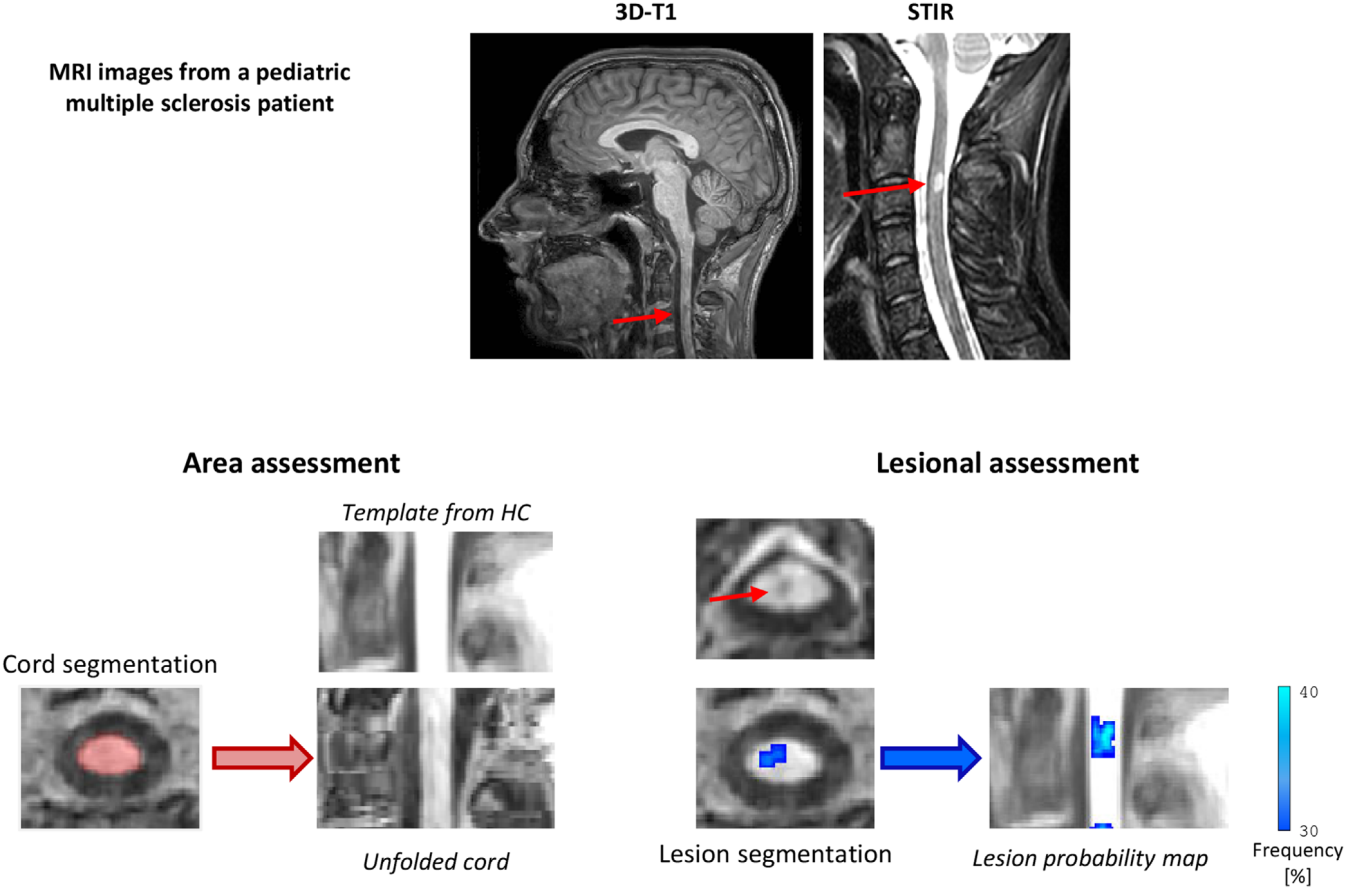
Spinal cord was segmented by using the active‐surface method from the most cranial section on which the odontoid process was visible to inferior border of C3 level (top row, in red). Unfolded spinal cord masks of pediatric MS patients were compared with spinal cord template obtained from healthy controls (HC) to detect spinal cord volumetric abnormalities. Spinal cord lesion segmentation (bottom row, in blue) was performed on brain 3D T1‐weighted images in each participant and unfolded T1‐hypointense lesion probability map was obtained.

After axial reformatting of brain 3D T1‐weighted scans, cord cross‐sectional area (CSA) was measured from the most cranial section on which the odontoid process was visible to the inferior border of the C3 level with the active‐surface method (Jim 7.0); CSA was then normalized for head size (nCSA) using the FSL SIENAx scaling factor, as previously described [10]. This approach has been validated in MS, with the active‐surface method achieving the best performance among different methods (Figure 1) [11].

### 2.8 Upper Cervical Cord Lesion Maps and Regional Volumetry Assessment

Unfolded upper cervical cord images of each subject were created by reformatting input images, together with corresponding T1‐hypointense lesion maps (whenever present), perpendicularly to the estimated cord center‐line [7]. Using the same procedure, cord contours were then used to produce binarized and unfolded upper cervical cord masks. Unfolded scans were rescaled in the through‐slice direction to the median cord length of HC (i.e., 37 mm) and coregistered to the mean cord image of HC, serving as cord template (using a scaling factor along the cranio–caudal direction) [7]. Finally, upper cord masks were smoothed using an anisotropic (1 × 1 × 2 mm^3^) Gaussian filter.

### 2.9 Statistical Analysis

Statistical analyses were performed using SPSS software, version 26 (SPSS, Chicago, IL). Demographic, clinical, and MRI features were compared between HC and pediatric MS patients (as a whole or divided according to the presence of cord lesions) using Fisher's exact test, Mann–Whitney *U* tests, or linear models, as appropriate.

Mean T1‐hypointense lesion probability maps were produced by averaging coregistered upper cord lesion masks. Voxel‐wise analysis of upper cord volumetry was performed using SPM12 full factorial models (sex‐, age‐, and total cord volume–corrected), first by comparing HC versus all pediatric MS patients, and then dividing patients according to the presence of cord lesions. Results were presented in terms of voxel count along the considered upper cord segment.

Correlations (*r*) of cord volumetry with disease duration and EDSS score were assessed using SPM12 multiple regression models corrected for sex, age, and total cord volume. Given the exploratory nature of this study, voxel‐wise results were thresholded at *p* < 0.001, uncorrected. Finally, correlations of T1‐hypointense cervical cord LV (log‐transformed) with EDSS score were also explored using SPSS partial Pearson's correlations corrected for age and sex.

## 3 Results

### 3.1 Demographic, Clinical, and Conventional MRI Findings

Compared to HC, pediatric MS patients had higher brain T2‐hyperintense WM LV (*p* < 0.001). No significant differences in terms of age and sex were observed (*p* ≥ 0.325). No upper cord lesions were detected in pediatric HC, whereas 12 out of 38 (32%) pediatric MS patients had ≥ 1 cervical cord lesion. Four patients had lesions spanning two levels, whereas one patient had a longitudinally extensive transverse myelitis. None of the lesions showed gadolinium enhancement on a post‐contrast T1‐weighted image (previously obtained on a recent clinical scan).

Compared to patients without cervical cord lesions, those with lesions were older (*p* = 0.007) and had a history of cervical myelitis (time from MRI acquisition = 0.5 [0.2;1.0] years). All patients with cervical myelitis experienced at least one clinical symptom related to the lesion. No differences in terms of sex, disease duration, EDSS score, treatment status, and brain T2‐hyperintense WM LV were observed (*p* ≥ 0.174) (Table 1).

**TABLE 1:** Main demographic, clinical, and MRI characteristics of healthy controls and pediatric multiple sclerosis patients.

Variable	HC (*n* = 13)	Pediatric MS (*n* = 38)	*p*	Patients without lesions (*n* = 26)	Patients with lesions (*n* = 12)	*p*
Sex						
Male (%)	4 (31)	10 (26)	0.734	5 (19)	4 (33)	0.423
Female (%)	9 (69)	28 (74)		21 (81)	8 (67)	
Median age (IQR) [years]	17.4 (15.0;17.8)	16.7 (14.9;17.3)	0.325	15.6 (13.4;16.9)	17.3 (16.9;17.7)	0.007
Median disease duration (IQR) [years]	—	0.7 (0.3;1.3)	—	0.6 (0.3;1.2)	1.0 (0.4;2.5)	0.174
Median EDSS score (IQR)	—	1.0 (1.0;1.5)	—	1.0 (0.0;1.5)	1.0 (1.0;1.5)	0.466
Patients receiving DMTs (%)	—	24 (63)	—	18 (69)	6 (50)	0.296
Median brain T2‐hyperintense WM LV^a^ (IQR) [mL]	0.0 (0.0; 0.1)	3.0 (2.7;3.5)	< 0.001	0.7 (0.3;2.7)	1.9 (0.6;3.7)	0.378
Median number of T1‐hypointense cervical cord lesions (IQR)	—	0 (0;1)	—	—	0 (0;1)	—
Median number of T2‐hyperintense cervical cord lesions (IQR)	—	0 (0;1)	—	—	0 (0;1)	—
Median T1‐hypointense cervical cord LV^a^ (IQR) [mL]	—	0.3 (0.1;0.4)	—	—	0.3 (0.1;0.4)	—
EM nCSA (SE) [mm^2^]	76.5 (2.3)	78.2 (1.5)	0.508	77.0 (2.1)	80.8 (2.7)	0.238

*Note:* Comparisons performed by Fisher's exact test (sex, number of lesions) and Mann–Whitney test (age, disease duration, EDSS score, WM lesion volumes). Age‐ and sex‐adjusted linear models were performed for normalized cross‐sectional area.

Abbreviations: DMTs, disease‐modifying therapies; EM, estimated mean; HC, healthy controls; IQR, interquartile range; LV, lesion volume; MRI, magnetic resonance imaging; MS, multiple sclerosis; nCSA, normalized cross‐sectional area; SE, standard error; WM, white matter.

^a^ T2‐hyperintense LVs were log‐transformed for the analyses.

### 3.2 Upper Cervical Cord Area and Volumetric Analysis

**FIGURE 2:**
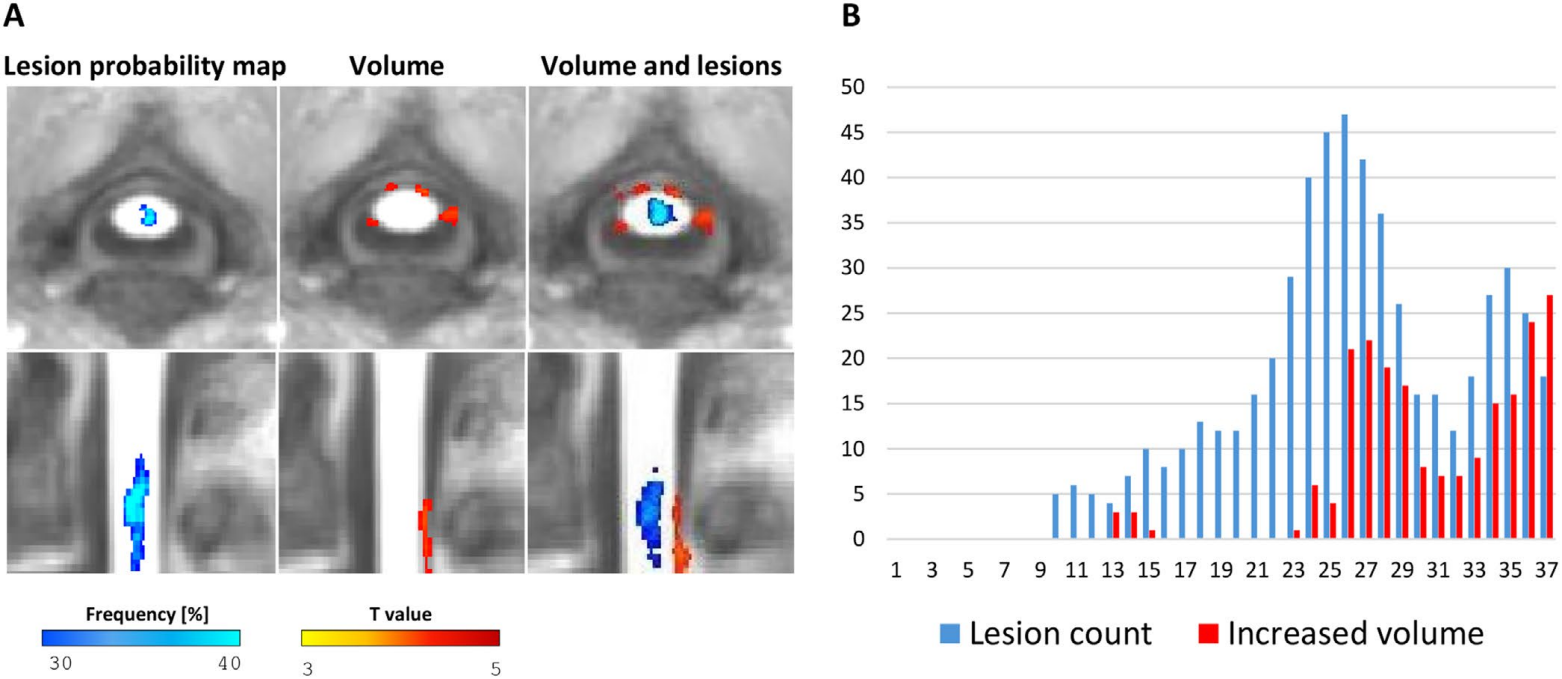
(A) T1‐hypointense lesion probability map and cervical spinal damage. Sagittal and axial views show T1‐hypointense lesion probability map at cervical level (left side, thresholded to retain only pixels having probability ≥ 30% of including lesions; blue color scale represents frequency values), sagittal and axial views show clusters of statistically significant increased cord volume versus healthy controls (HC) at cervical level (middle; color scale from yellow to brown indicates *t* value) and sagittal and axial views show relationship between lesion distribution and clusters of statistically significant increased volume (right side). (B) Bar graphs report voxel counts of T1 lesion volume and increased spinal cord volume along the cord axis within the considered cervical segment.

No nCSA difference was found between HC and pediatric MS patients (*p* = 0.508), nor in patients with lesions compared to those without lesions (*p* = 0.238) (Table 1). The voxel‐wise analysis showed no clusters of abnormal cord volumetry neither in pediatric MS patients versus HC, nor in pediatric MS patients without cord lesions versus HC. Clusters of increased volume were observed at C2–C3 level in patients with cervical cord lesions compared to both those without lesions and HC (*p* < 0.001, uncorrected, surviving at the conjunction analysis, *p* < 0.001). Along the cord axis, cord lesions were located in the posterior columns and tended to co‐localize with increased cord volume (Figure 2A,B).

### 3.3 Correlation Analysis

In pediatric MS patients with cord lesions, no associations were observed between regional clusters of increased cord volume, disease duration, and EDSS score. T1‐hypointense cervical cord LV did not associate with EDSS score (*r* = 0.476, *p* = 0.118).

## 4 Discussion

In this study, we evaluated a relatively large cohort of pediatric MS patients close to disease onset to characterize the extent of cervical spinal cord damage (in terms of lesions, area and volumetry) and its relationship with clinical disability.

We found that more than 30% of pediatric MS patients exhibited at least one cervical lesion, emphasizing the early and frequent spinal cord involvement in these patients [12, 13]. In line with a previous study [4], we did not observe nCSA differences between pediatric MS patients and HC. Interestingly, although no significant abnormalities in nCSA were observed in pediatric MS patients, independent of the presence of cord lesions, the voxel‐wise analysis revealed that MS patients with cord lesions had clusters of increased cord volume at C2–C3 levels compared to HC and patients without lesions. Although our results seem counterintuitive since spinal cord atrophy is expected especially in those patients having spinal cord lesions, it is noteworthy that previous studies performed in adult MS already showed a trend toward or a significant cord volume increase in clinically isolated syndrome [2] or RRMS [8] patients compared to HC. The increase in cord volume observed in our patients, characterized by a short disease duration (median = 0.7 years), may be related to the high amount of inflammation and oedema that typically characterize these patients close to disease onset. This hypothesis is supported by the trend toward co‐localization between lesions and cord volume increase. These processes may mask any destructive changes such as neuroaxonal loss, which occur from the earliest phases of the disease and would otherwise result in spinal cord volume loss [14].

Our analysis of the clinical relevance of spinal cord damage in pediatric MS patients with lesions showed no association between increased cord volume, disease duration, and EDSS score. This could be attributed to the limited range of disease duration and EDSS score observed in our cohort, as well as the repair capabilities, compensatory mechanisms, and neuroplasticity that characterize these patients [15, 16].

This study has some limitations. Due to the unavailability of high‐resolution cord sequences, we performed a partial analysis of the upper cord utilizing brain scans. However, considering the pediatric cohort explored, shorter MRI protocols may be particularly important. Indeed, acquiring spinal cord imaging can be challenging in pediatric patients, due to their limited tolerance for long examinations. As a result, spinal cord imaging is performed less frequently in both clinical practice and research settings compared to adult MS patients. Due to the difficulty in the enrolment of pediatric healthy subjects in MRI studies, we only included 13 HC, potentially limiting the extent of normal variability of spinal cord area and volumetry. Future studies should confirm our results on a larger cohort of patients and explore the spinal cord maturational processes occurring in pediatric MS compared to HC. Finally, we performed a cross‐sectional analysis of spinal cord area and volumetry. A longitudinal assessment may improve the understanding of the pattern of spinal cord damage accumulation in pediatric MS patients and its clinical relevance.

In conclusion, no upper cord atrophy was observed in pediatric MS patients. The regional volume increases seen in patients with lesions, likely reflecting inflammation, emphasize the inflammation‐driven characteristic of pediatric MS.

## Author Contributions

Concept and design: M.M., M.A.R., M.F. contributed to the conception and design of the study. Acquisition, analysis, or interpretation of data: M.M., P.V., P.P., M.R., M.G., L.M., M.A.R., M.F. contributed to the acquisition and analysis of data. Drafting of the manuscript: M.M., P.V., P.P., M.R., M.G., L.M., M.A.R., M.F. contributed to drafting the text and preparing the figures. Critical revision of the manuscript for important intellectual content: M.M., P.V., P.P., M.R., M.G., L.M., M.A.R., M.F. approved the final draft of the manuscript.

## Conflicts of Interest

The authors declare no conflicts of interest.

## Supporting information


Data S1.

## Data Availability

The dataset used and analyzed during the current study are available from the corresponding author on reasonable request.
