# Supplementary material for: Spinal Cord Abnormalities in Early Pediatric Multiple Sclerosis
Source: Ann Clin Transl Neurol. 2025 Apr 17;12(9):1901–6. doi: 10.1002/acn3.70046 (PMC12455864; doi:10.1002/acn3.70046)
Supplement: Supplementary file 1 — Data S1. [file ACN3-12-1901-s001.docx]

**Supplemental methods**

MRI acquisition. Using a 3.0 T Philips Ingenia MR scanner (Philips Medical System), the following sequences were acquired from all subjects during a single session: 1) Brain: a) three-dimensional (3D) fluid attenuated inversion recovery (FLAIR) (repetition time [TR]=4800 milliseconds [msec]; echo time [TE]=270 msec; inversion time [TI]=1650 msec; matrix size=256×256; field of view [FOV]=256×256 millimiters [mm]^2^; echo train length [ETL]=167; 192 contiguous sagittal slices, 1 mm thick); b) 3D T1-weighted turbo field echo (TR=7 msec; TE=3.2 msec; TI=1000 msec; flip angle=8°; matrix size=256×256; FOV=256×256 mm^2^; 204 contiguous sagittal slices, 1 mm thick); 2) Cord: a) sagittal 2D STIR (TR=2810 msec; TE=70 msec; TI=200 msec; ETL=14; matrix size=252×197; FOV=250×250 mm^2^; 16 contiguous sagittal slices, 2 mm thick).
